# Early Developmental Decline in HSP Expression Affects Subsequent Response to Transient Heat Exposure

**DOI:** 10.1093/iob/obaf046

**Published:** 2025-12-04

**Authors:** C R Warren, M B Wilken, J M Rollins, R T Paitz, R M Bowden

**Affiliations:** School of Biological Sciences, Illinois State University, Normal, IL 61790, USA; School of Biological Sciences, Illinois State University, Normal, IL 61790, USA; School of Biological Sciences, Illinois State University, Normal, IL 61790, USA; School of Biological Sciences, Illinois State University, Normal, IL 61790, USA; School of Biological Sciences, Illinois State University, Normal, IL 61790, USA

## Abstract

Understanding physiological responses to short-term changes in temperature is of growing interest considering the rising frequency and severity of transient temperatures such as heat waves. During the embryonic development of egg-laying vertebrates, inducible physiological responses to transient heat are likely critical to short-term survival but may also be energetically costly or disruptive to development. Inducible heat-shock proteins (HSPs) are conserved molecular chaperones which act to safeguard cellular protein homeostasis during transient stress. However, experiments in ectotherms have shown that overexpression of HSPs can increase embryonic mortality and reduce later thermotolerance. Yet, few studies have explored natural developmental patterns of *HSP* expression and heat inducibility in embryos of egg-laying ectothermic vertebrates. Using the red-eared slider turtle (*Trachemys scripta*), we characterized the response of five *HSP* genes in embryonic trunks following repeated 3-d transient heat wave exposures. Interestingly, we found that the expression of most *HSP*s naturally declined during early development and that warm temperatures amplified this decline, while also accelerating developmental rate. Only in a few instances did we observe induction of certain *HSP* genes during heat wave exposures, though this depended on the thermal history of the embryo. Specifically, induction of these genes during a particular heat wave was reduced in embryos that had already experienced two recent prior exposures relative to those experiencing it for the first or second time, suggesting repeated heat exposures can attenuate subsequent responses. The observed changes in *HSP* expression and inducibility may relate to an individual’s need to balance thermotolerance alongside extensive cellular differentiation and proliferation during early development. The effects of incubation temperature on these changes could also have important implications for how turtle embryos deal with subsequent heat stress and may be similarly present in other ectothermic vertebrates. Our study demonstrates the importance of considering ontogenetic changes in physiological responses to temperature even across embryonic development.

## Introduction

Short-term changes in environmental temperature have the potential to influence many facets of an organism’s biology and how they respond is therefore often critical to their survival and fitness (Angilletta Jr. 2009; Williams et al. 2016; Taff and Shipley 2023). Such responses can involve transient mechanisms of compensation (e.g., physiological, behavioral) and even long-term changes in phenotype (e.g., developmental, morphological). Given that many organisms routinely experience fluctuating transient temperatures in nature, intra- and inter-individual variation in trait responses to temperature shifts may partly define the ecological niche breadths and adaptive capacities of various species (Hutchinson 1957; Holt 2009; Carscadden et al. 2020). For egg-laying (oviparous) animals, such variation in inducible physiological responses are especially relevant during embryonic development when behavioral thermoregulation is absent (Booth 2006; Harada and Goto 2017; Noble et al. 2018; While et al. 2018; Gatto and Reina 2022). Such physiological responses are crucial for embryos to maintain or restore homeostasis following exposure to unusual or extreme transient temperatures (Williams et al. 2016; Burggren and Mendez-Sanchez 2023). This includes the timely regulation of molecular chaperones, osmolytes, immune factors, glucocorticoids, and/or states of cellular dormancy (Freedberg et al. 2008; Somero 2020; Vitousek et al. 2025). These responses, through their down-stream effects and energetic trade-offs, may also influence other complex developmental processes, such as those tied to differentiation, growth, and survival (O’Steen 1998; Feder and Hofmann 1999; O’Steen and Janzen 1999; Andrews et al. 2000; Ohmer et al. 2023). Moreover, repeated exposures to stressful thermal conditions could confer molecular memory within embryos, thereby altering physiological responses to subsequent exposures later in life (e.g., “heat-hardening”) (Paredes et al. 2016; Whitehouse et al. 2017; Alston et al. 2020; Moyen et al. 2020; Sessions et al. 2021; Jiang et al. 2024).

The unprecedented rise in global temperatures and increasing severity and frequency of anomalous transient temperatures such as heat waves are particularly notable for their negative consequences to organismal physiology, ecology, and life-history (Huey et al. 2012; Vasseur et al. 2014; Bestion et al. 2015; Shipley et al. 2020; Lauck et al. 2023; Taff and Shipley 2023). Regarding such transient temperatures, ectothermic vertebrates (e.g., fish, amphibians, and reptiles) are of special concern given that their body temperature and physiological functioning are dependent on ambient temperatures across all life stages, including embryonic development (Hall and Warner 2018, 2021; Jensen et al. 2018; Noble et al. 2018; Tanner et al. 2019; Feugere et al. 2021; Gatto and Reina 2022). In addition, developmental temperatures are known to elicit various persistent phenotypic effects in ectothermic vertebrates, such as sex, body size, metabolism, immunity, and behavior (O’Steen 1998; Freedberg et al. 2008; Jensen et al. 2018; Zhang et al. 2018; Tanner et al. 2019; Hall and Warner 2021; Ujszegi et al. 2022; De Jong et al. 2023; Ohmer et al. 2023). By characterizing epigenetic, physiological, and behavioral responses underlying acclimation to- or tolerance of- anomalous temperatures in ectotherms, researchers can better predict their sensitivity, adaptive capacity, and thus overall vulnerability to current or future climate change impacts (Churchill and Storey 1992; Packard and Packard 2001; Huey et al. 2012; Singh et al. 2020; Gatto and Reina 2022; Burggren and Mendez-Sanchez 2023).

**Fig. 1:**
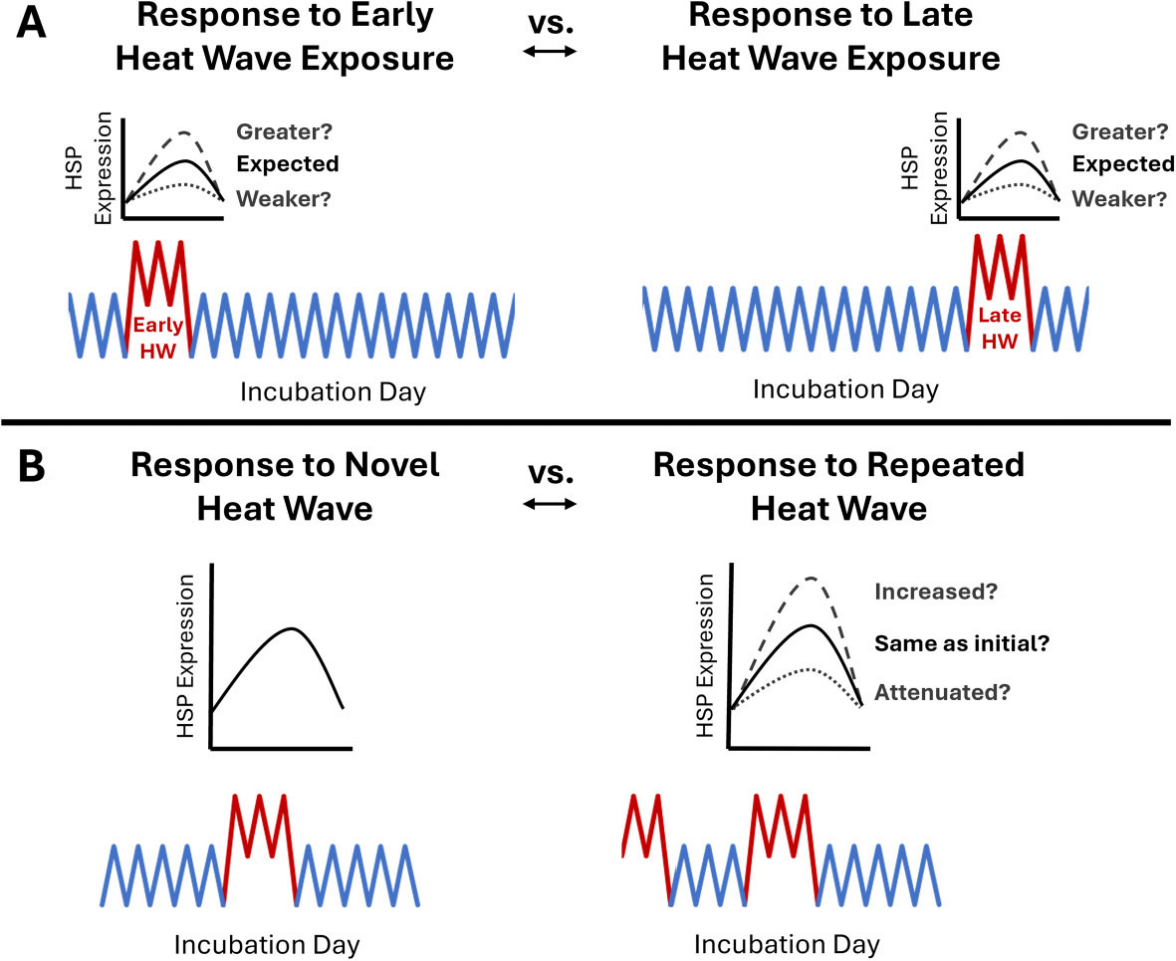
Potential differences in the response of inducible HSPs to transient heat wave (HW) exposure with (**A**) variation in exposure timing and hence embryonic stage (e.g., an early versus a late heat wave exposure) and (**B**) variation in the thermal history of the embryo leading up to the exposure (e.g., a novel versus a repeated exposure).

The heat-shock response (HSR) is an important and phylogenetically conserved mechanism of compensation induced during exposure to environmental stress, such as transient heat. This response is characterized by an upregulation in the group of molecular chaperones known as heat-shock proteins (HSPs) and a repression in the transcription of most other (non-chaperone) proteins to effectively reduce susceptible protein loads (Lindquist and Craig 1988; Moseley 1997; Sørensen 2010; Somero 2020; Hu et al. 2022). Under normal (unstressed) conditions, basally expressed HSPs assist in cell signaling, the transport, folding, and assembly of proteins, and are often thought to be necessary for normal development (Lindquist and Craig 1988; Walsh et al. 1993, 1999; Voss et al. 2000; Hawkins et al. 2008; Hu et al. 2022). During stressful conditions, induced HSPs act to safeguard cellular homeostasis by stabilizing native proteins, preventing toxic protein aggregates, and regulating apoptosis in response to insults such as oxidative damage (Moseley 1997; Beere et al. 2000; Jiang et al. 2011; Somero 2020; Lang et al. 2021 2021; Mengal et al. 2023). The adaptive significance of HSPs is further demonstrated by their conserved presence across prokaryotic and eukaryotic organisms and various life stages (Feder and Hofmann 1999; Sørensen 2010). For these reasons, intra- and inter-individual differences in the expression and thermal inducibility of HSPs likely have important implications for a species’ niche breadth and adaptive capacity (Kingsolver and Buckley 2020; Woodruff et al. 2022). Yet, such patterns of HSPs during embryogenesis, and how varying incubation conditions affect them, remain poorly studied for many oviparous ectotherms, particularly those of vertebrate taxa like reptiles. For example, the embryonic response of inducible HSPs during transient heat exposure in reptile embryos could vary with both the timing of exposure (and hence embryonic stage; Fig. 1A) and the thermal history leading up to the exposure (Fig. 1B) (Whitehouse et al. 2017; Sessions et al. 2021).

**Fig. 2:**
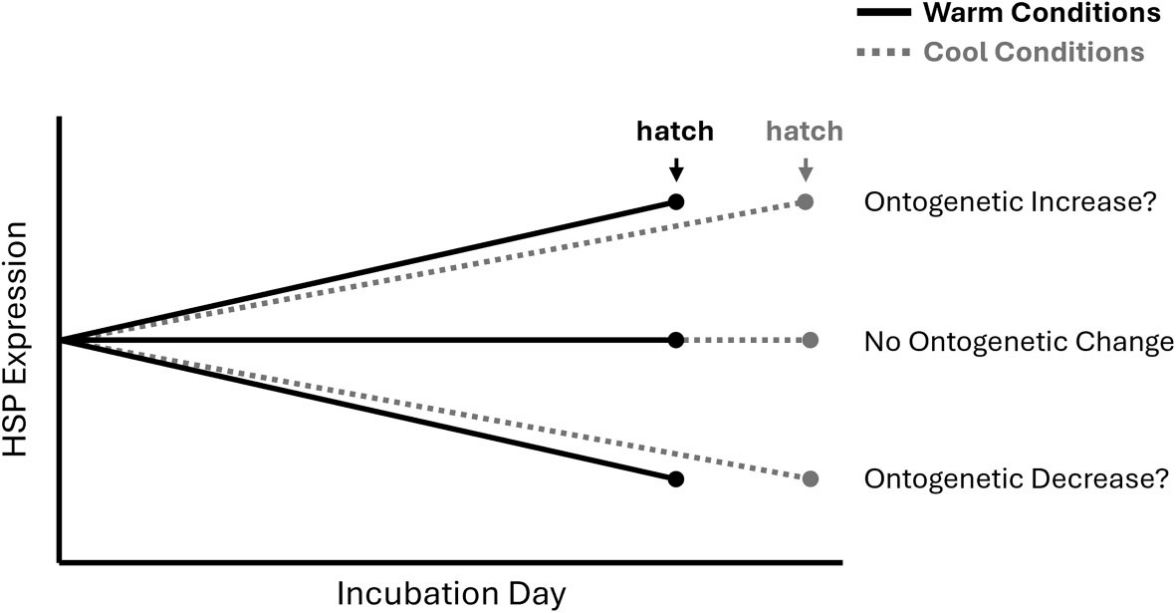
Potential variation in basal HSP expression patterns across embryonic development in the form of an ontogenetic increase or decrease in expression. Because warmer incubation conditions accelerate development (and therefore reduce incubation duration), we would expect that any ontogenetic change in HSP expression (if present) would occur sooner under warm conditions relative to cool conditions.

Early embryonic development is accompanied by extensive tissue differentiation and proliferation and, as a result, may be a period in which embryos are especially vulnerable to unstable thermal conditions and for which a highly reactive HSR is beneficial. Alternatively, a naturally (non-induced) elevated expression of HSPs early in development could function as a preemptive defense against environmental stressors and thus reduce the need for a highly reactive HSR, while simultaneously providing a surplus of chaperone machinery for cellular differentiation and tissue development (Prinsloo et al. 2009; Bradley et al. 2012; Miller and Fort 2018). Any ontogenetic changes in HSP expression should then have important implications for how embryos respond to transient temperature exposures across embryogenesis. Interestingly, temperature also affects the rate of development in ectotherms and therefore has the potential to influence the rate at which they experience an ontogenetic shift in HSP expression (Fig. 2). In this manner, the overall developmental patterns of HSP expression under naturally fluctuating temperatures could involve both direct temperature responses (Fig. 1) and ontogenetic-dependent changes in expression (Fig. 2).

Turtles are a group of reptiles that are often noted for their high risk to climate change, particularly exposures to heat. This is in part due to their life history (e.g., slow to reach sexual maturity), the presence of temperature-dependent sex determination (i.e., risk of sex ratio bias), and susceptibilities to other anthropogenic factors (e.g., pollutants, endocrine disruptors, etc.) (Poloczanska et al. 2009; Scott et al. 2012; Carter et al. 2018; Tanner et al. 2019; Paitz et al. 2022; Breitenbach et al. 2023; Warren et al. 2025). As ectotherms that lack parental care, embryonic development in turtles is also highly reliant upon their surrounding nest conditions (O’Steen 1998; Monaghan 2007; Bodensteiner et al. 2015). As such, it is likely that expression of HSPs in turtle embryos varies with overall incubation temperature which could then influence their ability to respond to subsequent transient heat.

Using a freshwater turtle model, we explored patterns of *HSP* expression and inducibility from whole embryonic trunk homogenates across a relatively early window of development (Table S1) following exposure to varying incubation temperatures, including simulated heat waves. Three separate studies were conducted across 2 years (one in 2023 and two follow-ups in 2024) using *Trachemys scripta*, a common model reptile in studies exploring the developmental consequences of incubation conditions. We chose to focus on five *HSP* genes (*HSP70A5, HSP70A8, HSP90AA1, HSP90B1*, and *HSPH1*) which appeared to increase in response to transient (5-days) heat exposure in a previous transcriptome that used gonads of late-stage *T. scripta* embryos (Marroquin-Flores 2022). Studies I and II investigated the effects of varying early transient heat wave exposures and study III compared early ontogenetic changes in *HSP*s between embryos maintained at cool or warm conditions. Our overall goals were to characterize the response of *HSP*s to transient heat wave exposure early in development and to subsequently explore the effects of ontogeny and thermal history on *HSP* expression in embryonic trunks. We initially hypothesized that the expression of *HSP*s would be stable across early development but increase during transient heat exposure. However, after the completion of study I, our hypothesis for studies II and III was that the expression of *HSP*s would decline during development, with declines being amplified by warmer incubation conditions.

## Methods

### Egg collection and incubation

**Fig. 3:**
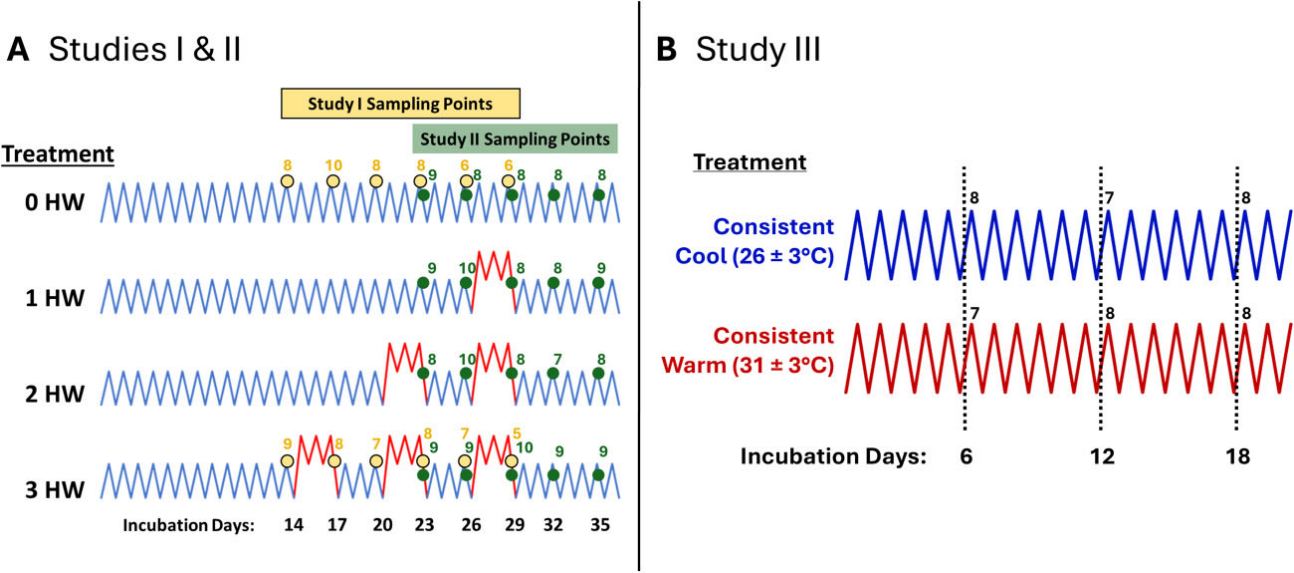
Treatment schematics and sampling plans for (**A**) studies I and II and (**B**) study III. Incubation treatments with sampling points for whole embryonic trunk tissues are denoted for study I (closed circles) in 2023, study II (open circles) in 2024, and study III (dashed black lines) in 2024. For (**A**) studies I and II, cool baseline (26 ± 3°C) and heat wave (HW; 31 ± 3°C) conditions are depicted as lower blue and upper red zigzag lines, respectively. For (**B**) study III, embryos were held consistently at either cool (top) or warm (bottom) conditions throughout incubation. The number of resulting embryos acquired per sampling is listed above each sampling point.

In both study years, *T. scripta* eggs were collected and shipped on their day of oviposition from Concordia Turtle Farm, LLC (Jonesville, LA, USA) to the laboratory at Illinois State University. Eggs were shipped overnight in 2023 but were delayed for 24 h in 2024. Upon arrival, eggs were immediately numbered and systematically split across predetermined treatment groups and sampling dates to help control clutch effects. We then placed eggs into plastic boxes containing moist vermiculite wherein they were maintained at −150 kPa throughout incubation. Baseline conditions consisted of cool fluctuating temperatures (26 ± 3°C) using incubators programmed to fluctuate between their minimum and maximum temperatures on a 12:12 h schedule (IPP 110 Plus, Memmert GmbH + Co.KG, Schwabach, Germany). We chose this fluctuating thermal regime as it was within the bounds of expected naturalistic thermal conditions during cooler summer days in Illinois (USA) between the months of June and July when *T. scripta* typically lay their first clutch of eggs (Carter et al. 2018). Transient (3-d) heat waves were introduced by moving experimental eggs to incubators consisting of warm fluctuating temperatures (31 ± 3°C) programmed on a 12:12 h schedule, as these conditions approximate temporary bouts of high summer temperatures observed in wild *T. scripta* nests in Illinois and are near the high thermal limit of *T. scripta* embryos (Les et al. 2009; Breitenbach et al. 2020). Temperature exposures differed across studies and treatment groups (see Fig. 3). Because warmer incubation conditions increase developmental rate, we also assessed the embryonic stage of several embryos in each treatment group using the staging criteria of Greenbaum (2002). For study I, embryos were staged only on the final sampling day, whereas staging was done across sampling in studies II and III. None of our studies had embryos which surpassed stage 20 (out of 26) which is just before roughly half of the total duration of embryonic development (Greenbaum 2002; Lin et al. 2024). As such, we refer to our studies collectively as covering a large window of early development (see Table S1 for more details). However, it is worth noting that our observed stages cover the “early” and “middle” phases of development according to Lin et al. (2024).

### Study I: three repeated transient heat exposures

In 2023, experimental eggs underwent three repeated 3-d transient heat waves, each separated by 3-d back at baseline conditions. The first of these heat waves occurred between incubation days 15 and 17, the second between days 21 and 23, and the third between days 27 and 29. Movements of experimental eggs in between cool and warm incubators occurred roughly between 1200 and 1300 CDT when incubators were near their minimum daily temperatures (23°C for cool, 28°C for warm). Therefore, eggs experienced an immediate change of 5°C when they were moved in between cool and warm incubators. Control eggs remained at baseline conditions throughout incubation. Example thermal traces captured every 90-min from iButton temperature loggers (Maxim Integrated, Wilmington, MA, USA) within our experimental and control egg boxes are shown in Supplementary Figure S1. A subset of 5–10 embryos per treatment were sampled (details below) on incubation days 14, 17, 20, 23, 26, and 29, corresponding to just before experimental eggs were moved to and from the heat wave conditions (Fig. 3A, 2023 sampling points). For embryos staged during the day 29 sampling, we observed control embryos between stages 16 and 17 and experimental embryos between stages 18 and 19, suggesting that the three repeated heat waves prompted a 1-to-2-stage advancement in development.

### Study II: varying repeated transient heat exposures

Here, experimental eggs underwent a varying number of multiple 3-d transient heat waves: once between days 27–29 (1 HW), twice between days 21–23 and 27–29 (2 HW), or three times between days 15–17, 21–23, and 27–29 (3 HW). All experimental groups therefore experienced their final exposure between days 27–29, but differed in that this was either their 1st, 2nd, or 3rd exposure (Fig. 3A). Control eggs remained at cool baseline conditions throughout incubation (0 HW). Moving experimental eggs in between cool and warm incubators in study II differed slightly from study I but still consisted of an immediate change of 5°C. Movements of experimental eggs from cool to warm incubators (i.e., to onset heat wave conditions) occurred at approximately 0900 CDT when incubators were near their mean daily temperatures (from ∼26 to 31°C), whereas movements of eggs from warm to cool incubators (i.e., returning to baseline) occurred at approximately 1430 CDT when incubators were near their maximum daily temperatures (from ∼34 to 29°C) (Fig. S2). A subset of 7–10 embryos per treatment were sampled (details below) on days 23, 26, 29, 32, and 35 which spanned the final heat wave exposure of experimental eggs and 5 days after (Fig. 3A, 2024 sampling points). In terms of embryonic stages, we observed an average 1-to-2-stage advancement for embryos of the 3 HW group (stages 16–19) relative to the 0 HW group (stages 15–17) across the sampling window. From days 29 to 35, the embryos of the 1 HW and 2 HW groups generally followed the stages of the 0 HW and 3 HW groups, respectively.

### Study III: ontogenetic changes under consistent cool or warm conditions

Here, we investigated whether *HSP* expression changes across a 12-day window of early development in embryos incubating under more consistent thermal conditions and whether this differs between embryos held at our cool or warm thermal regimes. Eggs were maintained at either 26 ± 3°C or 31 ± 3°C which correspond, respectively, with the baseline and heat wave conditions used during the first two studies (Figure S2). Note that all eggs in study III were topically treated with 10 μL of 70% ethanol on incubation day 1 as they had additionally served as “controls” for a separate study in which they were compared to eggs treated with estrogens (not reported here). To capture potential ontogenetic changes in *HSP* expression across a relatively large window of early development, a subset of 7–8 eggs per incubation treatment were sampled (details below) on incubation days 6, 12, and 18 (Fig. 3B). Embryos were of visibly similar stages for both warm and cool treatments on day 6 which we termed “pre-stage 13.” On incubation day 12, cool treatment embryos had remained at “pre-stage 13”, while the warm treatment had advanced to stage 13, and on day 18, the average stages for the cool and warm treatment embryos were 14.5 and 17, respectively.

### Tissue collection and processing for gene expression

Across each study, whole embryonic trunk tissues were sampled from a subset of embryos per sampling point (Fig. 3) and stored in 1 mL of TRIzol^TM^ Reagent (Invitrogen, Carlsbad, CA, USA) at −80°C until later ribonucleic acid (RNA) extraction. Freshly thawed trunks were homogenized using a pestle tissue homogenizer after which RNA was extracted using chloroform and isopropanol and subsequently diluted in 200–300 µL of molecular grade water. We determined RNA concentrations for each sample using a NanoDrop^TM^ 2000c (Thermo Scientific, Waltham, MA, USA) and further diluted them to 125 ng/µL. Next, complementary DNA (cDNA) was synthesized from 1 µg of RNA per sample using the Maxima First Strand cDNA Synthesis Kit with dsDNase (Thermo Scientific, Waltham, MA, USA). Resulting cDNA samples were each diluted 1:9 in UltraPure^TM^ distilled water (Invitrogen, Carlsbad, CA, USA) and used for real-time quantitative polymerase chain reaction (RT-qPCR) to obtain expression of our five *HSP* genes of interest and two housekeeping genes, glyceraldehyde-3-phosphate dehydrogenase (*GAPDH*) and elongation factor 1 alpha (*EF1α*). The primer sequences for the target *HSP*s and housekeeping genes used in this study were designed using the publicly available *T. scripta elegans* genome (Simison et al. 2020). For each gene, diluted cDNA samples were loaded in triplicate on 384-well plates with PowerUp^TM^ SYBR^TM^ Green Master Mix (Applied Biosystems, Foster City, CA, USA) and processed using QuantStudio 7 Real-Time PCR System Software (Applied Biosystems, Foster City, CA, USA). Normalized expression of each *HSP* was obtained with the 2^−ΔCT^ method using either *GAPDH* or *EF1α* as the housekeeping gene depending on the study (Livak and Schmittgen 2001; Rao et al. 2013; Breitenbach et al. 2020). We found that the cycle threshold (CT) values of *EF1α* were more stable across sampling days than those of *GAPDH* for study III only. Although variation in *GAPDH* was observed across sampling days for studies I and II (Fig. S3), we later discuss why this variation is unlikely to explain our major patterns of normalized *HSP* expression in these studies.

### Statistical analysis

All statistical analyses were conducted in R (R Project for Statistical Computing, Vienna, Austria). In all studies, we tested the effect of treatment, incubation day, and their interaction on the normalized expression of each of the *HSP*s using separate linear models and a significance cutoff of *P* < 0.05. Logarithmic or square root transformations were often used to improve the fit of gene expression data to a normal distribution. In study III, a Gamma distribution was instead specified only for the model on *HSP90B1* due to transformation failing to normalize its positively skewed expression data. When significant interactions were identified from a linear model, we performed *post hoc* comparisons between (1) incubation days within each treatment and (2) between treatments at each incubation day, using the false discovery rate (FDR) adjustment for multiple testing. However, if the interaction term was non-significant in the original model, we dropped it and re-tested the model for main effects only. Significant main effects of treatment or day were followed with *post hoc* comparisons between treatments averaged across days or days averaged across treatments, respectively, again utilizing FDR adjustment.

## Results

### Study I: a natural decline in *HSP* expression is amplified by repeated heat

**Fig. 4:**
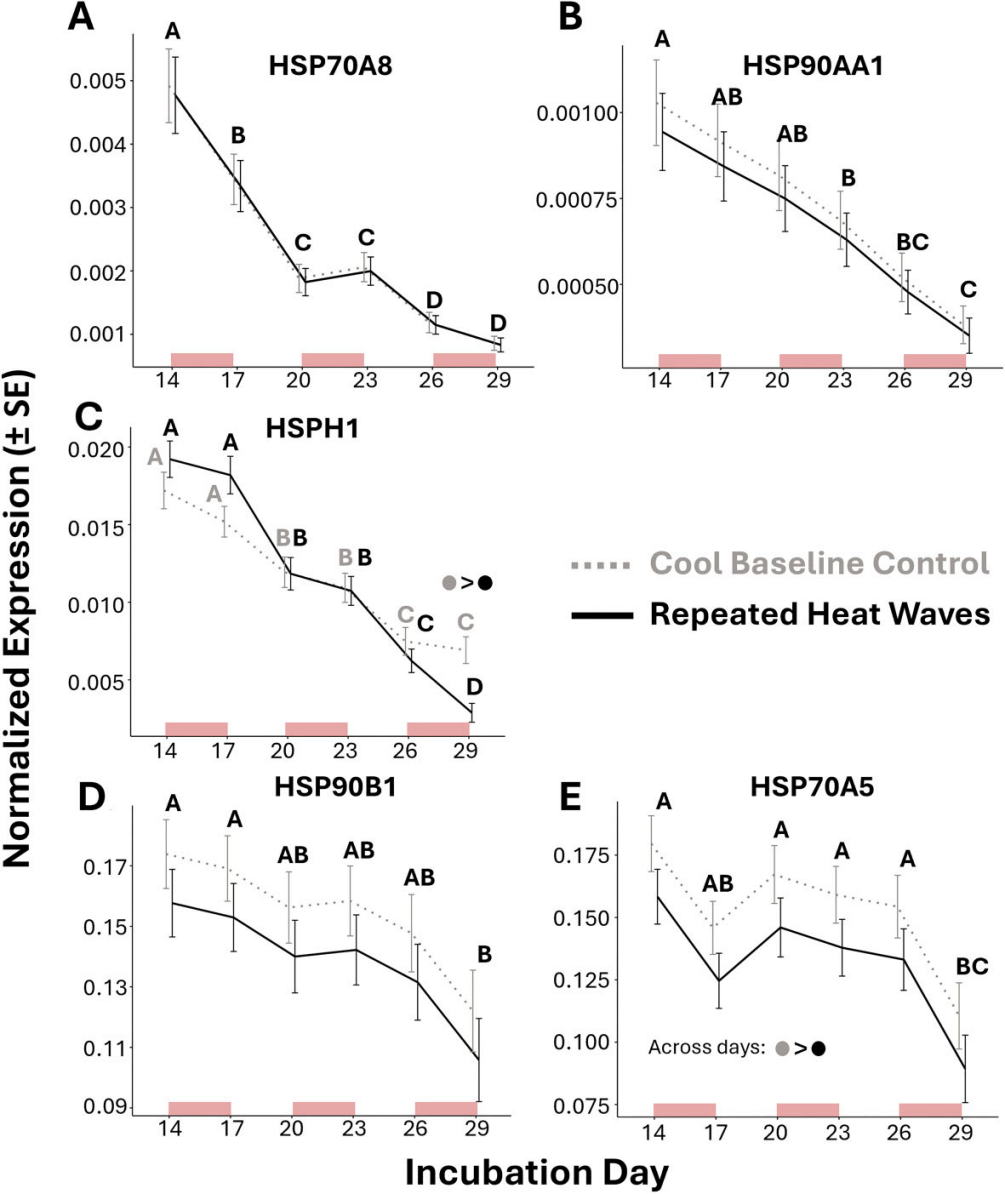
Study I results indicate the significant effects of repeated heat wave exposure and incubation day on the estimated marginal means (±SE) of normalized *HSP* gene expression in red-eared slider turtle embryos. Dotted gray and solid black lines represent expressions at cool baseline (control) and repeated heat wave exposed treatments, respectively. Days that do not share a letter overhead are statistically different from one another. Note that the differences between days are within each treatment for *HSPH1* (C) due to a significant interaction between treatment and day for this gene, while HSP70A8 (A), HSP90AA1 (B), HSP90B1 (D) and HSP70A5 (E) are averaged across the two treatments due to there being a lack of interaction. Differences between treatments in (C, E) are represented by dots colored to match their treatment group, with the group preceding the “>” sign having higher expression than the one following. Light blocks along the *x*-axis denote the timing at which 3-d heat wave exposures (31 ± 3°C) occurred for embryos of the repeated heat wave treatment from otherwise cool baseline conditions (26 ± 3°C).

The results from study I linear models investigating the effects of treatment, day, and their interaction for each gene are provided in Table 1. We initially predicted that (1) the expression of *HSP*s would increase during each 3-d heat wave exposure and return to pre-exposure levels following 3-d back at baseline temperatures, and (2) the shape of this response would change with accumulating repeated exposures (i.e., altered inducibility). For *HSP70A8, HSP90AA1, HSP90B1*, and *HSP70A5*, linear models revealed no significant interaction between treatment and day. After removing the interaction term, only the main effect of day remained significant for these genes except for *HSP70A5* which also had a significant effect of treatment (Table 1). The *post hoc* comparison between days averaged across treatments indicated several significant declines in these *HSP*s across the sampling window (Fig. 4). Expression of *HSP70A5* was also generally lower in experimental relative to control embryos (*P* < 0.05). For *HSPH1*, the interaction term for treatment and day was significant (Table 1), with the *post hoc* comparisons between treatments at each day showing lower expression in the experimental group relative to controls only on the final sampling day (*P* < 0.001). *Post hoc* comparisons between days in each treatment also showed that *HSPH1* significantly declined in both treatment groups between days 14–20 (*P* < 0.01) and 20–26 (*P* < 0.01) (Fig. 4C).

**Table 1:** The linear model results concerning the effects of treatment, day, and their interaction on the normalized expression of our target heat-shock protein genes from study I (see Fig. 3A for treatment and sampling details).

	Study I: Linear model results								
	Treatment effect			Day effect			Interaction term		
	*df*	*F value*	*P*	*df*	*F value*	*P*	*df*	*F value*	*P*
HSP70A8	*1*	0.11	0.74	*5*	30.55	**<0.001**	*5*	0.21	0.96
HSP90AA1	*1*	0.81	0.37	*5*	9.04	**<0.001**	*5*	0.96	0.45
HSPH1	*1*	1.41	0.24	*5*	56.79	**<0.001**	*5*	4.18	**<0.01**
HSP90B1	*1*	3.24	0.08	*5*	2.43	**<0.05**	*5*	2.17	0.07
HSP70A5	*1*	5.74	**<0.05**	*5*	4.09	**<0.01**	*5*	1.42	0.23

*Note:* Note that, in cases where the interaction term was non-significant, the remaining results shown for treatment and day are after its removal from the model.

Significant *P*-values are in bold.

### Study II: repeated exposures alter *HSP* inducibility to subsequent heat

**Fig. 5:**
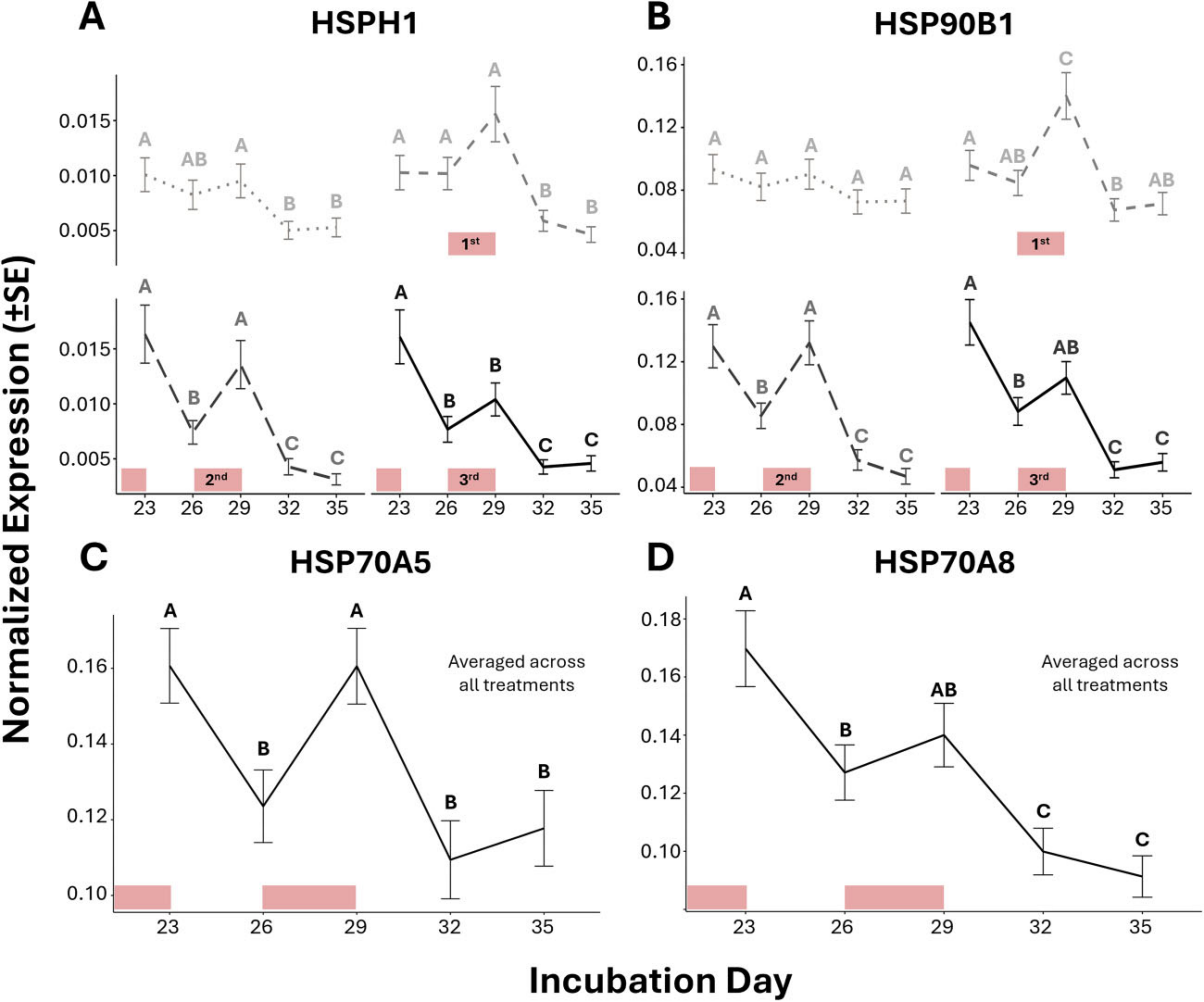
Study II results indicate the significant effects of varying multiple HW exposures and incubation day on the estimated marginal means (±SE) of normalized *HSP* gene expression in red-eared slider turtle embryos. Expression at 0 HW (dotted, light gray), 1 HW (dashed, gray), 2 HW (long dash, dark gray), and 3 HW (solid black) are shown as separate panels for both *HSPH1* (A) and *HSP90B1* (B) given significant interactions between treatment and day for these genes. Meanwhile, *HSP70A5* (C) and *HSP70A8* (D) expressions are shown as single (black) lines representing the average across all 4 treatments at each day due to a lack of interaction and only a main effect of day for these genes (but see Supplementary Figure S4 for all treatments). For each gene (and within each treatment group), days that do not share a letter overhead are statistically different. Comparisons between treatments are not depicted. Light blocks along the *x*-axis denote the timing at which 3-d HW exposures (31 ± 3°C) occurred for embryos of the heat wave treatments from otherwise cool baseline (26 ± 3°C) conditions (see Fig. 3A for details).

Table 2 provides the linear model results of study II concerning the effects of treatment, day, and their interaction for each gene. Owing to the results of study I, we predicted here that the expression of *HSP*s would be highest at our earliest sampling points and decline thereafter, with more heat exposures resulting in greater declines by the end of sampling. From their models, we found no significant interaction between treatment and day for either *HSP70A5* or *HSP70A8* and, after dropping the interaction term, only a main effect of day remained for both genes (Table 2). The *post-hoc* analyses between days averaged across treatments revealed an overall decline in both *HSP70A5* and *HSP70A8* across this study window (Fig. 5C and D). Significant interactions between treatment and day were, however, observed in the models of both *HSP90B1* and *HSPH1* (Table 2). The *post hoc* analyses comparing days within each treatment revealed several significant declines in *HSP90B1* and *HSPH1* across the sampling window within various treatments, including the 0 HW group for *HSPH1*. However, both *HSP90B1* and *HSPH1* also significantly increased during the final heat wave exposure for certain experimental treatments. An increase in *HSP90B1* was observed for both the 1 HW and 2 HW groups (*P* < 0.01 for both), but not the 3 HW group (*P* > 0.1) (Fig. 5B), while an increase in *HSPH1* was observed for only the 2 HW group (*P* < 0.01), but neither the 1 HW or 3 HW groups (*P* > 0.05 for both) (Fig. 5A). Only *HSP90B1* had significant differences when comparing treatments on each day, in which it was lower in the 0 HW and 1 HW groups relative to 3 HW on day 23 (both *P* < 0.05), lower in the 0 HW relative to the 1 HW and 2 HW groups on day 29, and higher in the 0 HW and 1 HW groups relative to 2 HW on day 35 (*P* < 0.05 for all). Lastly, for the model on *HSP90AA1*, we found no significant interaction nor main effects of treatment or day (Table 2).

**Table 2:** The linear model results concerning the effects of treatment, day, and their interaction on the normalized expression of our target heat-shock protein genes from study II (see Fig. 3A for treatment and sampling details).

	Study II: Linear model results								
	Treatment effect			Day effect			Interaction term		
	*df*	*F value*	*P*	*df*	*F value*	*P*	*df*	*F value*	*P*
HSP70A8	*3*	2.25	0.08	*4*	10.45	**<0.001**	*12*	0.82	0.62
HSP90AA1	*3*	2.27	0.08	*4*	1.67	0.16	*12*	1.4	0.17
HSPH1	*3*	0.98	0.40	*4*	41.07	**<0.001**	*12*	1.99	**<0.05**
HSP90B1	*3*	0.60	0.62	*4*	37.37	**<0.001**	*12*	3.57	**<0.001**
HSP70A5	*3*	1.83	0.14	*4*	5.97	**<0.001**	*12*	0.99	0.46

*Note:* Note that, in cases where the interaction term was non-significant, the remaining results shown for treatment and day are after its removal from the model.

Significant *P*-values are in bold.

### Study III: the expression of some *HSP*s diverge early between embryos of warm and cool incubation conditions

**Fig. 6:**
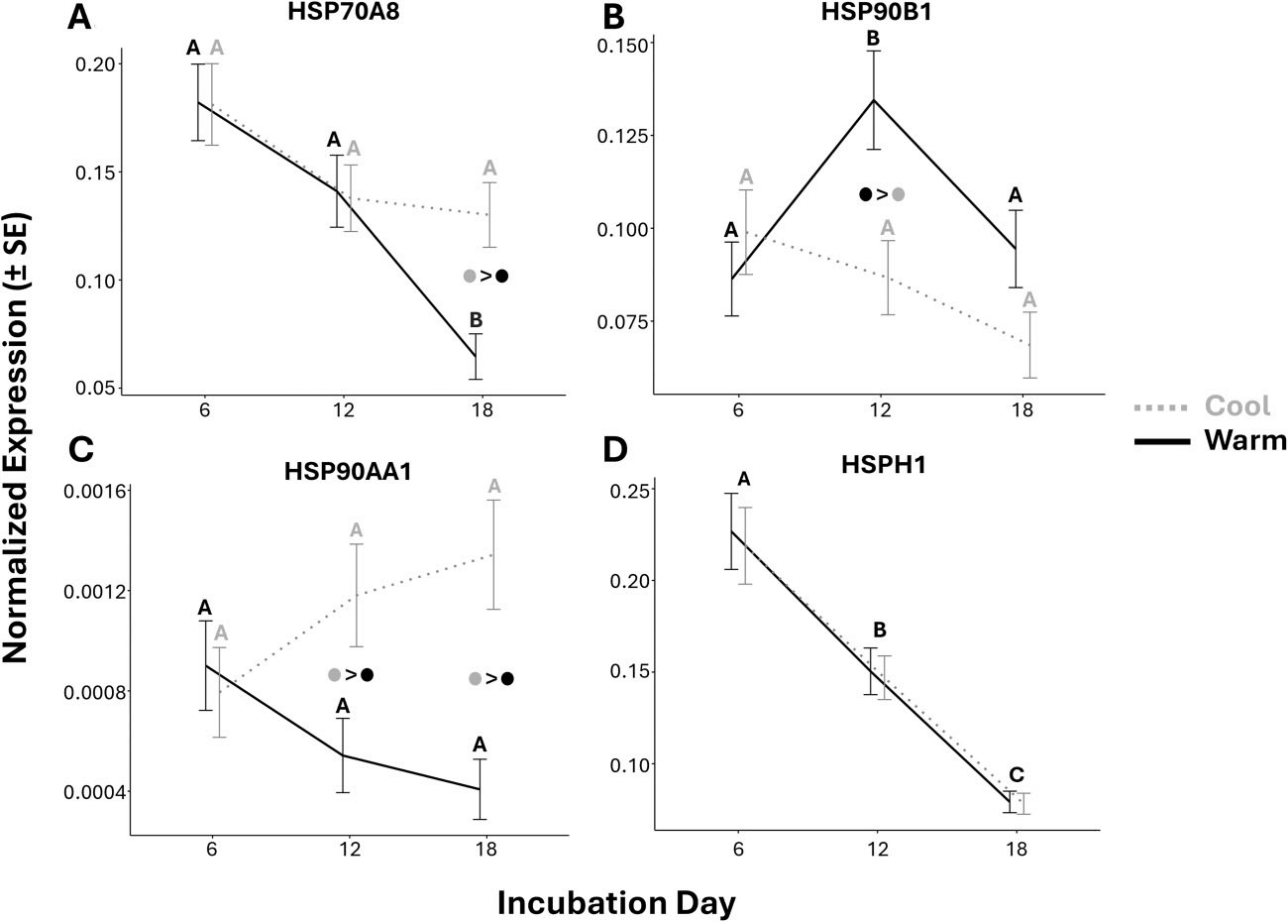
Study III results indicate the significant effects of incubation temperature (consistent cool (26 ± 3°C; dashed gray line) conditions versus consistent warm (31 ± 3°C; solid black line)) and incubation day (6, 12, and 18) on the estimated marginal means (±SE) of normalized *HSP* gene expression in red-eared slider turtle embryos. Days that do not share a letter overhead are statistically different from one another. Note that the differences between days are within each treatment except for *HSPH1* (D), which is averaged across the two treatments due to there being a lack of interaction and only a significant effect of day for this gene. Significant differences between treatments at a given day are represented by dots colored to match their treatment group in (A–C), with the group preceding the “>” sign having higher expression than those following.

Results of the study III linear models are provided in Table 3 with the effects of treatment, day, and their interaction for each gene. We predicted here that the expression of most *HSP*s would diverge between embryos of the cool and warm treatments. Specifically, we expected *HSP*s to be higher in those under cool conditions by our final sampling point (incubation day 18) as the result of a more pronounced decline in the warm treated embryos. First, the model for *HSPH1* indicated no significant interaction and only a main effect of day but not treatment (Table 3). A *post hoc* analysis for *HSPH1* comparing days averaged across treatments indicated that average normalized expression steadily dropped over two-fold between days 6 and 18 (*P* < 0.001; Fig. 6D). However, significant interactions between incubation treatment and day were found for *HSP70A8, HSP90AA1*, and *HSP90B1* (Table 3). From *post hoc* analyses, we found that the warm incubation treatment group significantly decreased in *HSP70A8* across sampling (*P* < 0.001) (Fig. 6A). However, the warm treatment also experienced an increase in *HSP90B1* between days 6 and 12 (*P* < 0.05), which then returned to near original expression levels by day 18 (Fig. 6B). Within the cool treatment group, no significant changes in *HSP70A8, HSP90AA1*, or *HSP90B1* were observed across sampling. From the comparisons of treatment at each given day, we found that the warm group had lower expression of *HSP90AA1* on days 12 (*P* < 0.05) and 18 (*P* < 0.001), lower *HSP70A8* on day 18 (*P* < 0.001), and higher *HSP90B1* on day 12 (*P* = 0.01) (Fig. 6A–C). Lastly, the model for *HSP70A5* was non-significant, with no effects from the interaction, nor main effects of treatment or day after dropping the interaction (Table 3).

**Table 3:** The linear model results concerning the effects of treatment, day, and their interaction on the normalized expression of our target heat-shock protein genes from study III (see Fig. 3B for treatment and sampling details).

	Study III: Linear model results								
	Treatment effect			Day effect			Interaction term		
	*df*	*F value*	*P*	*df*	*F value*	*P*	*df*	*F value*	*P*
HSP70A8	*1*	3.79	0.06	*2*	15.96	**<0.001**	*2*	4.53	**<0.05**
HSP90AA1	*1*	11.88	**<0.01**	*2*	0.03	0.97	*2*	4.78	**<0.05**
HSPH1	*1*	0.09	0.76	*2*	50.73	**<0.001**	*2*	0.47	0.63
HSP90B1	*1*	5.23	**<0.05**	*2*	3.58	**<0.05**	*2*	3.74	**<0.05**
HSP70A5	*1*	0.04	0.84	*2*	0.16	0.85	*2*	0.07	0.93

*Note:* Note that, in cases where the interaction term was non-significant, the remaining results shown for treatment and day are after its removal from the model.

Significant *P*-values are in bold.

## Discussion

**Fig. 7:**
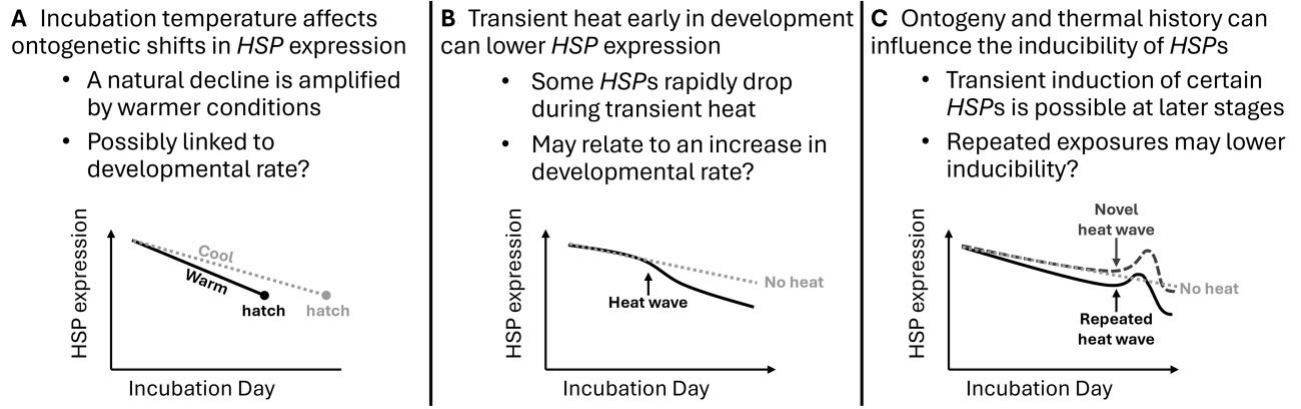
Synopsis for the potential effects of (**A**) overall incubation temperature, (**B**) transient heat exposure, and (**C**) ontogeny and thermal history (e.g., past heat wave exposure) on the expression or inducibility of *HSP* genes during early embryonic development of red-eared slider turtles (*Trachemys scripta*) based on the major findings of our three studies.

In all three studies, we observed natural declines in *HSP* expression within whole embryonic trunks during early development. Heat exposure often amplified this decline resulting in heat exposed embryos exhibiting reduced *HSP* expression on certain incubation days relative to those incubated at overall cooler conditions (Fig. 7A and B). We suspect that as heat speeds up development, the decline in *HSP*s is also accelerated, thereby causing the decrease in *HSP*s to occur sooner for heat exposed embryos. Indeed, our three repeated heat wave treatments in studies I and II, as well as 18-days at warm conditions in study III, resulted in the advancement of roughly 2-stages by the end of sampling relative to baseline control embryos. For the few instances where we observed evidence of *HSP* induction by heat exposure in embryonic trunks, developmental stage and thermal history appeared to be important influential factors (Fig. 7B and C).

The results of study I, counter to our original predictions, suggested that the repeated 3-d heat wave exposures during this early period of development did not induce *HSP* expression. Instead, these exposures generally appeared to speed up a natural decline in *HSP*s relative to control embryos. We saw a similar decline in the expression of these *HSP*s in study II but also found evidence of context-dependent induction by heat wave exposure for *HSP90B1* and *HSPH1*. Specifically, these two genes were temporarily induced by the final heat wave, but only when it was the first or second exposure and never the third, suggesting that repeated exposures may lower inducibility to subsequent exposures (Fig. 7C). The results of study III also demonstrated that the expression of *HSP*s is dependent on overall incubation temperature and provided further evidence of a natural decline that, at least for some genes, occurs quicker or more prominently under warmer conditions. Therefore, while we see that certain *HSP*s can be induced by heat during certain developmental contexts, the most striking finding across our three studies is that *HSP*s are declining in whole trunks early in development, with warmer conditions possibly speeding up the decline.

Together our results indicate a phenomenon in *T. scripta* embryos that has, to our knowledge, not yet been reported for any ectothermic vertebrate: a natural decline in *HSP* gene expression across early embryonic development which is amplified by heat (Fig. 7A and B). Given that our initial aim was to explore induced expression of *HSP*s in response to transient heat exposure early in turtle development, we were surprised to instead find an overall developmental decline in expression. Consequently, this finding should be an important point of consideration for any future studies attempting to similarly characterize HSP responses in ectothermic vertebrate embryos. It is possible that these *HSP*s are naturally elevated very early in embryonic development in association with a high prevalence of undifferentiated tissues, and that their expression naturally declines as development progresses (thus faster at warmer conditions). In addition, left-over maternal HSP mRNA transferred prior to oviposition might partially explain an initially elevated and subsequently declining expression of *HSP*s in early embryos (Lockwood et al. 2017; Lim and Bernier 2022), although such maternal transfer of *HSP*s remains to be thoroughly demonstrated in reptiles.

A naturally elevated expression of *HSP*s early in development could offer *T. scripta* embryos several benefits, including an enhanced tolerance to environmental perturbations and the necessary chaperone machinery for extensive tissue differentiation. It is interesting to consider the consequences of the observed decline in *HSP*s across early development which may also correspond with an increase in their inducibility to transient heat. Perhaps these changes are tied to the energetic trade-offs associated with chaperone proteins, and an embryo’s need to therefore properly balance expression for the purposes of tissue protection, differentiation, and/or growth. For example, a naturally elevated expression of HSPs might protect the embryo from stressful stimuli during its earliest stages of development without the need for their induction in response to a stressor, similar to the “constitutive frontloading” of HSPs observed in some sessile marine organisms (Dong et al. 2008; Barshis et al. 2013; Collins et al. 2023). Low heat inducibility of *HSP*s during this early window of development could then prevent further induction on top of an already elevated expression profile and is possibly the result of self-inhibition (i.e., negative feedback). In addition, avoiding the need for HSR activation early in development could help prevent its transcriptional repression of other non-chaperone proteins which may otherwise be crucial to early tissue formation. As development proceeds, a natural decline in the constitutive expression of *HSP*s and an associated increase in their inducibility might favor energy conservation by permitting *HSP* upregulation only as needed (e.g., during transient heat). Assuming warmer incubation conditions accelerate the decline in *HSP*s leaves us questioning what trade-offs are associated with this variation. Specifically, what consequences are associated with shorter versus longer periods of a naturally elevated and declining expression of *HSP*s, and do these declines occur in similar fashion between the various tissues comprising whole trunks? We suspect that such variation could, for example, influence an organism’s susceptibility to oxidative damage during subsequent stress and/or affect long-term thermotolerance.

In terms of the response of *T. scripta* embryos to transient heat waves, certain *HSP* genes appeared to exhibit plastic responses which varied with thermal history and perhaps ontogeny (Fig. 7C). The effects of thermal history were observed in study II, in which repeated heat exposures seemed to have lowered the inducibility of certain *HSP*s to subsequent exposures. Similar attenuation of the HSR by repeated transient heat exposures have been reported in the embryos of whitefish (*Coregonus clupeaformis*) (Whitehouse et al. 2017; Sessions et al. 2021). The potential effects of ontogeny, on the other hand, may help explain why studies I and II differed in their evidence of induction in response to transient heat. Recall that no induction was observed in study I, which covered an earlier window of development than study II. We suspect that inducibility could be weakest at our earliest sampling days due to *HSP* expression already being naturally elevated and actively declining, with inducibility increasing as the decline approaches a lower baseline. The finding that repeated heat waves reduce inducibility of *HSP*s also possibly explains why, in study I, we still found no evidence of induction even during the 3rd heat wave, as by this time they had already experienced two recent exposures. Like the varying rates of *HSP* decline, these changes to inducibility could have consequences for embryonic tolerance to subsequent heat exposures.

Changes to *HSP* inducibility following recent repeated heat exposures could be linked to proposed developmental trade-offs of the HSR, with costs including the repression in global protein production and energy requirements of HSP functioning (Feder and Hofmann 1999; Sørensen 2010; Gao et al. 2014; Tedeschi et al. 2015; Somero 2020). One of the first studies to demonstrate such a trade-off was in fruit fly (*Drosophila melanogaster*) larvae, in which experimentally increasing the copies of HSP70 genes via mutation, despite strengthening induction of HSP70 during 1 h exposures to heat, resulted in decreased developmental rate and increased mortality (Krebs and Feder 1997). In terms of reptiles, an overexpression of HSP70 in soft-shelled turtle (*Pelodiscus sinensis*) embryos achieved through plasmid injection conferred increased tolerance to heat exposure as late-staged embryos but appeared to reduce heat tolerance in resulting hatchlings (Gao et al. 2014). A study on the velvet gecko (*Amalosia lesueurii*) also found that hatchlings from hotter nests exhibited reduced heat and cold tolerance relative to hatchlings from cooler nests, for which the authors speculate possible underlying HSP-related trade-offs (Dayananda et al. 2017). The induction of HSPs during heat exposure may therefore be adaptive but also costly for ectothermic embryos: offering short-term protection from the damaging effects of heat but at high levels capable of disrupting development and reducing later thermotolerance. In addition, an aberrantly high expression of HSPs is hallmark to many human diseases, including certain cancers and autoimmune disorders (Murphy 2013; Hu et al. 2022; Singh et al. 2024), which may represent other negative consequences associated with their overexpression in vertebrates. The existence of trade-offs such as these might explain our observed attenuated inducibility of certain *HSP*s with recurring transient heat in *T. scripta* embryonic trunks.

Regarding gene differences, although *HSP90B1* and *HSPH1* were found to be inducible by our naturalistic heat exposures in certain contexts, *HSP70A8* and *HSP90AA1* appeared non-induced during these exposures. These latter genes might therefore maintain low heat inducibility across early turtle development. In particular, *HSP70A8* appeared to steadily decline in all studies, suggesting the protein product of this *T. scripta* gene may function similarly to its purported ortholog, heat-shock cognate 70 (HSC70), which is constitutively expressed and critical to embryogenesis in zebrafish (Wang et al. 2022). Concerning the variation in *GAPDH* observed in studies I and II (Fig. S3), we have reason to believe that this does not explain our observed patterns of normalized *HSP* expression in these studies for at least two reasons. First, despite the increases in *GAPDH* which should correspond with an increase in the resulting normalized expression of *HSP*s across those days, we instead observed an overall decline in the normalized expression of most *HSP*s. In fact, this rise in *GAPDH* might partially obscure the true extent of this natural decline in *HSP*s. Second, even in cases where *HSP*s increased in response to the final heat wave for certain treatments in study II, the control (0 heat wave) group remained stable in *HSP* expression across those days despite the rise in *GAPDH*, meaning *GAPDH* is unlikely to explain the increases observed in those experimental groups. Nonetheless, identifying a more stable housekeeping gene across these early periods of *T. scripta* embryonic development would be beneficial for future studies using whole trunk homogenates.

In conclusion, we find that incubation temperature and ontogeny have important effects on the expression of *HSP* genes in *T. scripta* embryos and their inducibility to transient heat (Fig. 7). Our three studies together demonstrate that (1) early *T. scripta* embryos experience an overall natural decline in *HSP* expression which can be amplified by heat and (2) the inducibility of *HSP*s to heat exposure depends on developmental stage and thermal history. It is unclear whether these changes in inducibility are long-lasting, though this is likely dependent on the underlying mechanism(s). For example, inhibitory negative feedback by HSPs on their own transcription may be more transient than epigenetic modifications to gene responsiveness. The fitness implications associated with this temperature-dependent variation in *HSP* expression and inducibility are interesting questions left to explore, and may involve trade-offs to thermotolerance, growth, and survival. Given that only whole trunks were studied here, potential tissue specificity in *HSP* expression and inducibility warrants further investigation. Indeed, such tissue differences should be particularly relevant to ectothermic vertebrates given that transient temperatures can influence an array of developmental processes and trigger persistent effects on phenotype (Carter et al. 2018; Hall and Warner 2018; Breitenbach et al. 2020; Sessions et al. 2021; Ujszegi et al. 2022; De Jong et al. 2023; Ohmer et al. 2023; Warren et al. 2025). This includes the temperature-dependent differentiation of gonads in some species, of which sexually dimorphic gonadal expressions of certain *HSP*s (or upstream heat-shock factors) have been identified in some species, suggesting potential roles of heat-shock chaperones in the temperature-driven development of sex (Kohno et al. 2010; Furukawa et al. 2019; Liu et al. 2023). Finally, future studies involving repeated sampling across exposures of shorter (e.g., hours within a single day) and longer (>3 days) heat exposures, perhaps of varying intensities, could allow for more complete characterizations of *HSP* inducibility than the simple response metric used here.

Given the risks posed to ectotherms by climate change-driven transient temperatures, understanding how individuals of a species respond to anomalous exposures should help inform us of their susceptibility and potential needs for intervention. With the growing number of studies exploring intraspecific variation of physiological responses to naturalistic and variable temperatures, it should be worthwhile to include vulnerable periods of development when behavioral thermoregulation is absent or highly limited. However, when it comes to the HSR, our work here suggests that factors such as ontogeny and recent thermal history can have major impacts on *HSP* expression and inducibility during embryonic development and should therefore be considered when attempting to measure HSRs during development. The natural decline in *HSP*s during early embryonic development observed here in *T. scripta* might similarly exist in other oviparous ectotherms and have important implications for their fitness across diverse and changing environments.

## Supplementary Material

obaf046_Supplemental_File

## Data Availability

Data available from the Dryad Digital Repository: https://doi.org/10.5061/dryad.zs7h44jnx.
