# Supplementary material for: Early Developmental Decline in HSP Expression Affects Subsequent Response to Transient Heat Exposure
Source: Integr Org Biol. 2025 Dec 4;7(1):obaf046. doi: 10.1093/iob/obaf046 (PMC12754417; doi:10.1093/iob/obaf046)
Supplement: obaf046_Supplemental_File [file obaf046_supplemental_file.docx]

| **Stage** | **Important Morphological Developments** | **Approximate % of Embryonic Development** |
| --- | --- | --- |
| **“Pre-13”** | Turtle eggs are laid at the late gastrula stage, resemble a typical early vertebrate body plan by stage 7, and from stages 7 – 12, develop early forelimb buds, melanistic eyes, liver tissue, and an enlarged cranium | **0 – 20 %** |
| **13 – 14** | Progressive eye (pupil), upper and lower jaws, and advancing forelimb bud-to-paddle development | **21 – 30 %** |
| **15 – 16** | Progressive lower jaw development, appearance of forelimb pigmentation and digits (ridges), an early carapace ridge develops | **31 – 36 %** |
| **17 – 18** | Caruncle appears as the beak develops on the upper jaw, further digit development (from serrations to distinct digits) on all limbs, lower eyelid appears, developing marginal and vertebral scutes appear on the carapace | **37 – 41 %** |
| **19 – 20** | Further progression of the lower eyelids, the digits are increasingly distinct and elongated, all developing scutes are present on the carapace and plastron, slight pigmentation appears across the developing carapace | **42 – 50 %** |

**Table S1.** Important morphological developments and the approximate percentage of embryonic development (relative to the total incubation duration) completed for each of the observed stages within our studies. Morphological characteristics corresponding to each stage are adapted directly from Greenbaum (2002), while approximate percentages of development are based on estimates from Lin et al. (2024) and our own observations.


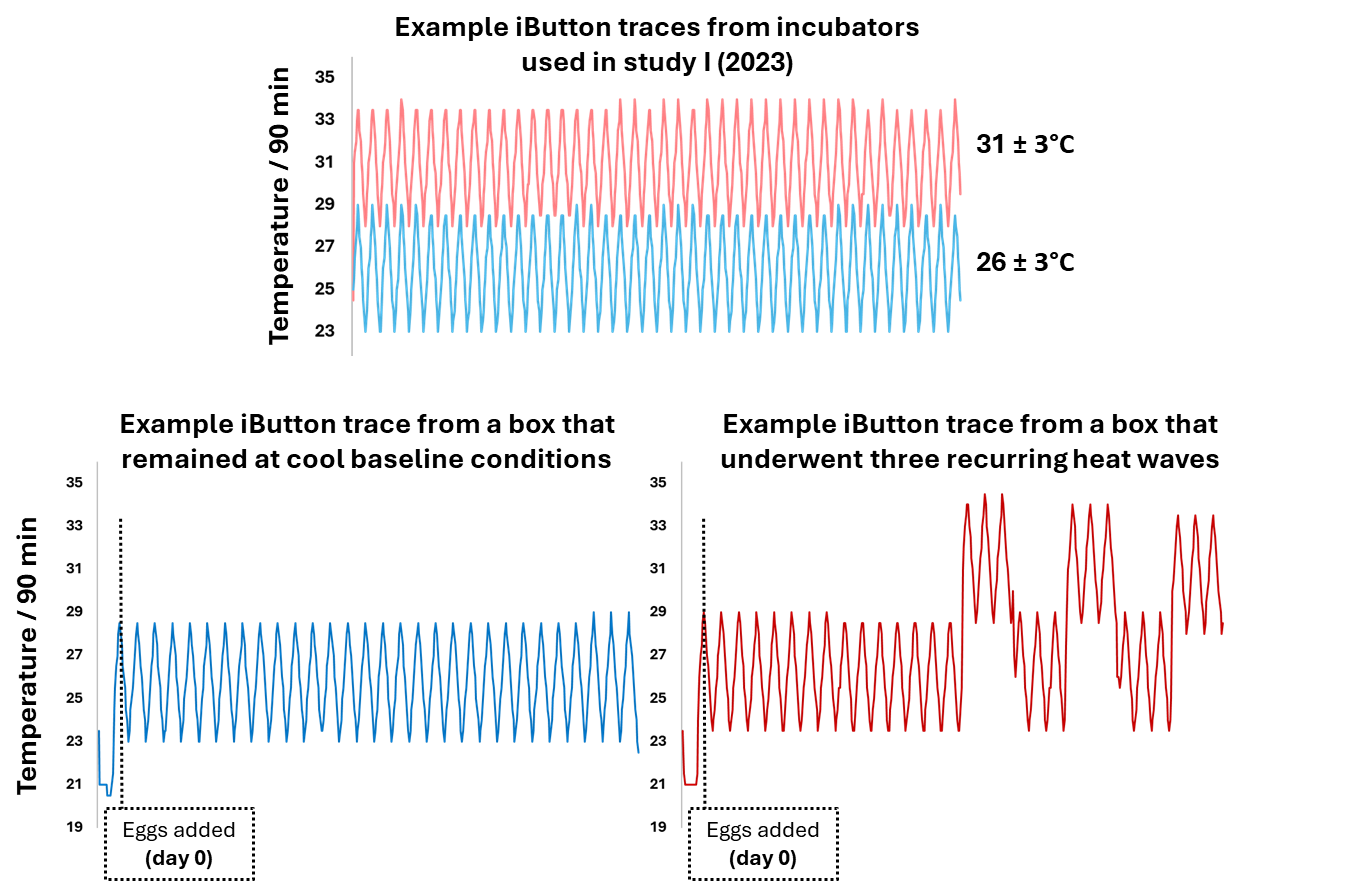


**Figure S1.** Example iButton thermal trace data from **study I** incubators (top) set to warm (red) and cool (blue) conditions, and egg boxes (bottom) that stayed at cool control conditions (bottom left; blue) or moved between cool and warm incubators to simulate three recurring heat waves (bottom right; red).


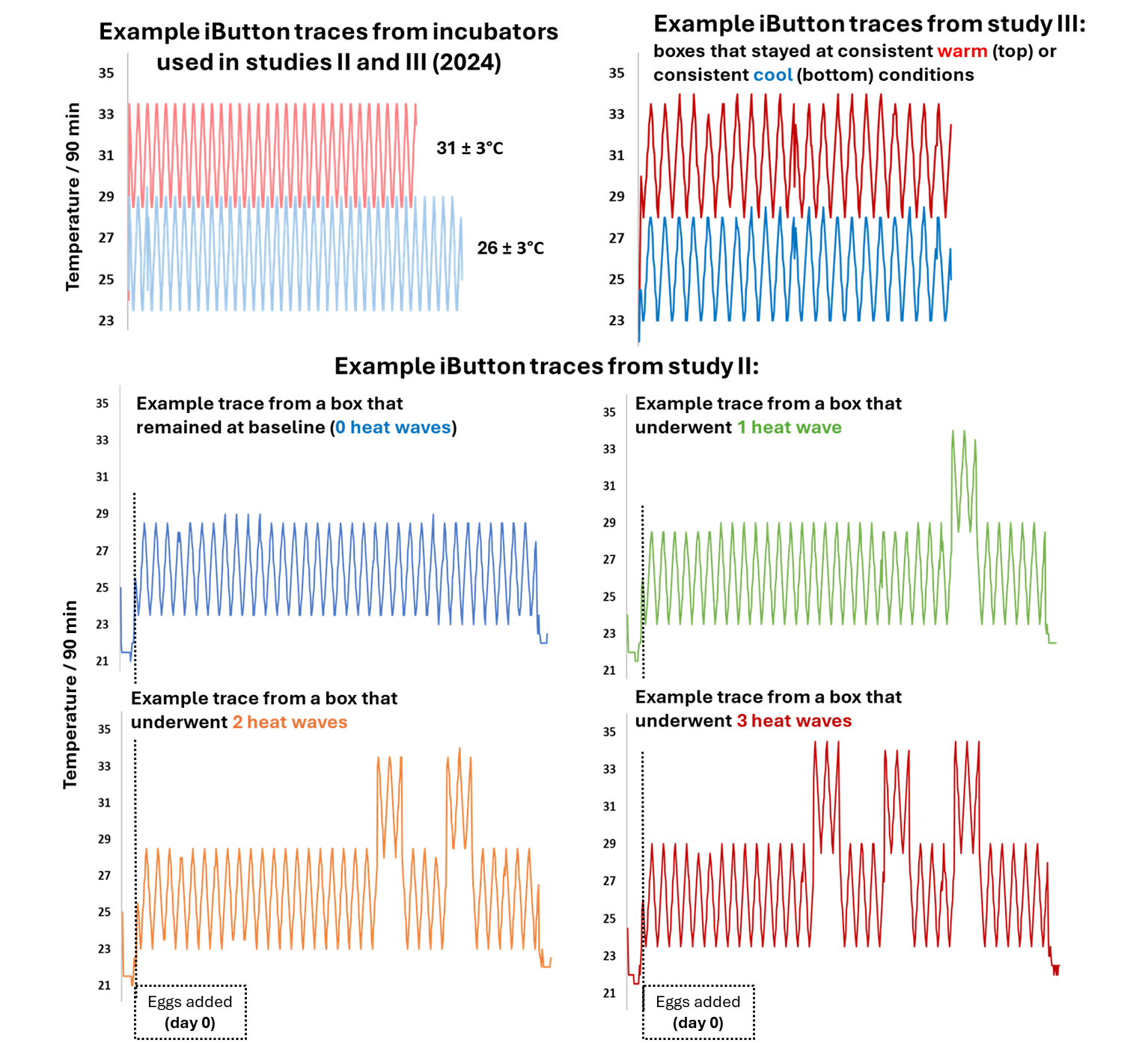


**Figure S2.** Example iButton thermal trace data from **studies II and III**. The incubators (top left) were set to warm (red) and cool (blue) conditions. Study III egg boxes (top right) remained consistently at either cool conditions (blue) or warm conditions (red). Study II egg boxes (bottom 4 panels) either stayed at cool control conditions (0 heat waves; blue) or experienced 1- (green), 2- (yellow), or 3- (red) simulated heat wave exposures.


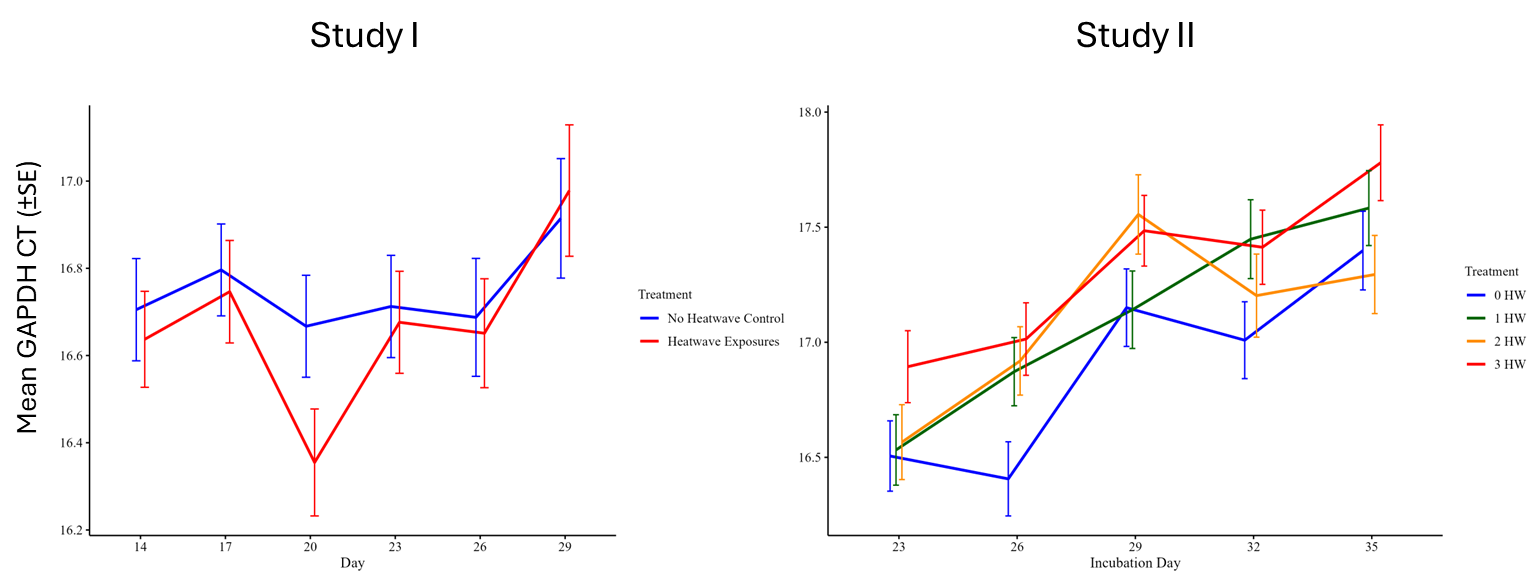


**Figure S3.** The mean *GAPDH* CT (±SE) for study I (left) and study II (right) demonstrate the variation in this housekeeping gene across sampling days. Reasoning for why this variation is unlikely to explain our assessed patterns of normalized heat-shock protein gene expression from these studies is provided in the main text’s discussion section.

**
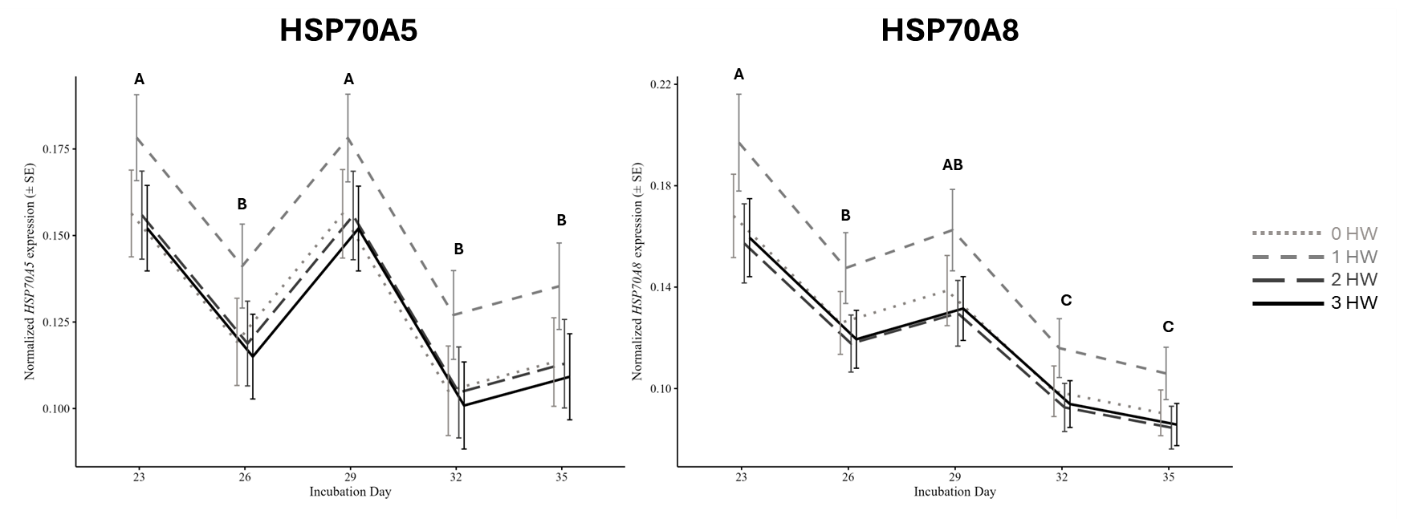
**

**Figure S4.** The estimated marginal means (±SE) for *HSP70A5* (left) and *HSP70A8* (right) from study II showing here all four heat wave (HW) treatment groups (0 HW, 1 HW, 2 HW, and 3 HW). Treatments were found to be statistically similar across sampling for these two genes and only a main effect of day was found. Days that do not share a letter overhead are statistically different from one another (averaging across treatments).
